# Supplementary material for: Effects of a New Combination of Natural Extracts on Glaucoma-Related Retinal Degeneration
Source: Foods. 2021 Aug 15;10(8):1885. doi: 10.3390/foods10081885 (PMC8391439; doi:10.3390/foods10081885)
Supplement: Supplementary file 1 [file foods-10-01885-s001.zip › foods-1317007-supplementary.pdf]

Supplementary Material

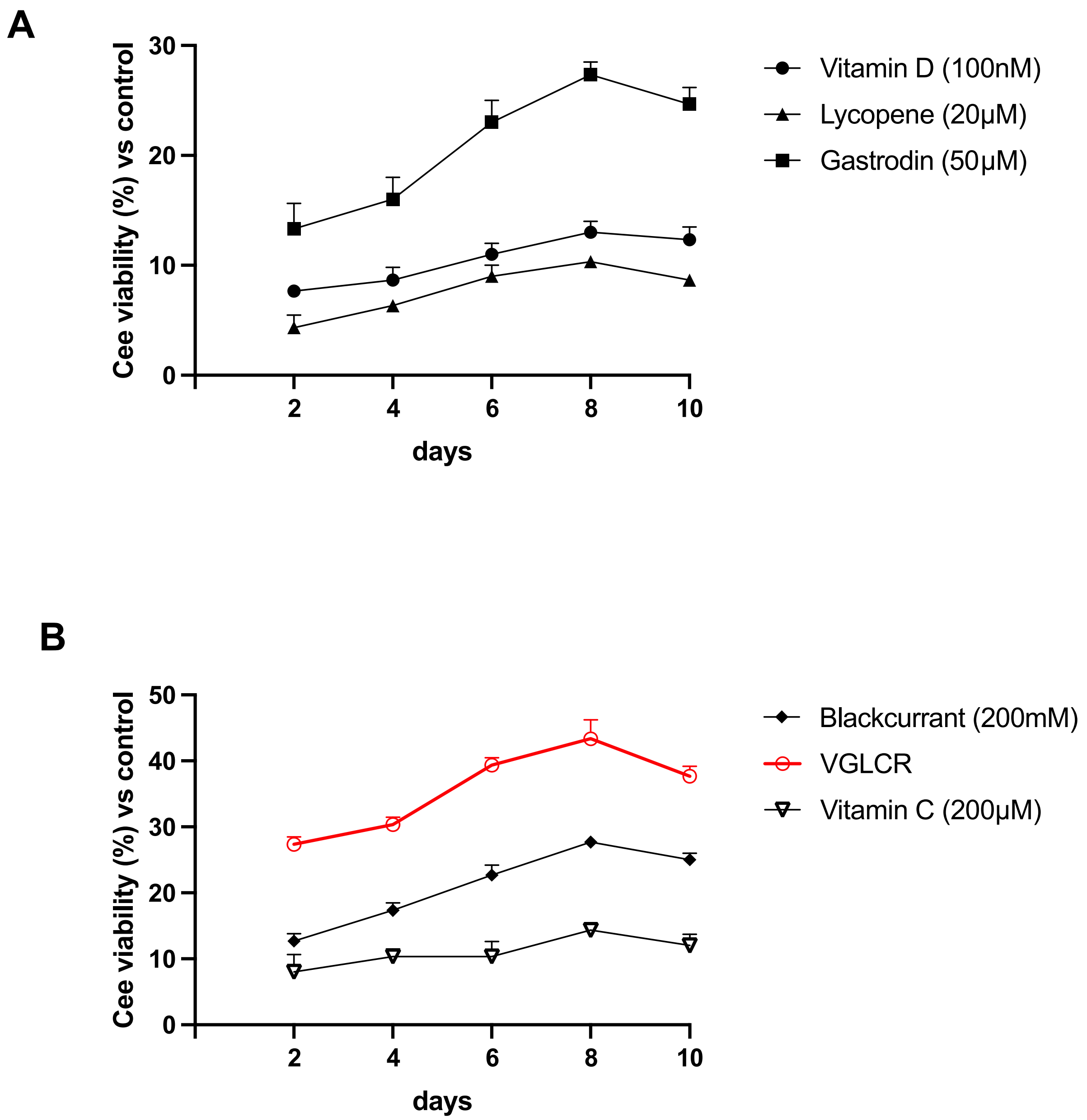

Supplementary Figure S1. Cell viability on RGCs analyzed for 10day checked each 2days of stimulation. In (A), time-course study of 100nM Vitamin D, 20µM Lycopene and 50µM gastrodin on cell viability. In (B), time-course study of 200µM vitamin C, 200mM blackcurrant and VGLCR= (V=vitamin D + G=gastrodin + L=Lycopene + C=vitamin C + R=Blackcurrant) on cell viability. The results are expressed as means±SD (%) vs control (0% line) of 4 independent experiments each performed in triplicate.
